# Supplementary material for: End-stage renal disease accompanied by mild cognitive impairment: A study and analysis of trimodal brain network fusion
Source: PLoS One. 2024 Jun 13;19(6):e0305079. doi: 10.1371/journal.pone.0305079 (PMC11175492; doi:10.1371/journal.pone.0305079)
Supplement: Attachment — Submitted filename: Response to Reviewers.docx [file pone.0305079.s001.docx]

**Journal Requirements:**

1. Please ensure that your manuscript meets PLOS ONE's style requirements, including those for file naming. The PLOS ONE style templates can be found at https://journals.plos.org/plosone/s/file?id=wjVg/PLOSOne_formatting_sample_main_body.pdf and https://journals.plos.org/plosone/s/file?id=ba62/PLOSOne_formatting_sample_title_authors_affiliations.pdf

Reply: It has been checked and corrected.

2. Please note that PLOS ONE has specific guidelines on code sharing for submissions in which author-generated code underpins the findings in the manuscript. In these cases, all author-generated code must be made available without restrictions upon publication of the work. Please review our guidelines at https://journals.plos.org/plosone/s/materials-and-software-sharing#loc-sharing-code and ensure that your code is shared in a way that follows best practice and facilitates reproducibility and reuse.

Reply: We can provide it if necessary.

3. Thank you for stating the following financial disclosure:

"Top Talent of Changzhou “The 14th Five-Year Plan” High-Level Health Talents Training Project (grant No. 2022CZBJ072)."

Please state what role the funders took in the study. If the funders had no role, please state: "The funders had no role in study design, data collection and analysis, decision to publish, or preparation of the manuscript."

If this statement is not correct you must amend it as needed.

Please include this amended Role of Funder statement in your cover letter; we will change the online submission form on your behalf.

Reply: Funders Haifeng Shi: Topic selection design, paper review.( As explained in the cover letter)

4. In this instance it seems there may be acceptable restrictions in place that prevent the public sharing of your minimal data. However, in line with our goal of ensuring long-term data availability to all interested researchers, PLOS’ Data Policy states that authors cannot be the sole named individuals responsible for ensuring data access (http://journals.plos.org/plosone/s/data-availability#loc-acceptable-data-sharing-methods).

Reply: Contact information for the Ethics Committee: 2392058632@qq.com/15804942480

5. Please review your reference list to ensure that it is complete and correct. If you have cited papers that have been retracted, please include the rationale for doing so in the manuscript text, or remove these references and replace them with relevant current references. Any changes to the reference list should be mentioned in the rebuttal letter that accompanies your revised manuscript. If you need to cite a retracted article, indicate the article’s retracted status in the References list and also include a citation and full reference for the retraction notice.

Reply: It has been checked and modified.

**Additional Editor Comments:**

1)In introduction you state “Investigating the relationship between changes in brain functional networks and MCI in patients with ESRD is an area of active research interest [17]” …Reference [17] has no mention of ESRD?

Reply: Reference [17] has been replaced.

2)You introduce “mode of brain network” with this sentence “The utilization of multimodal fusion methods can offer more comprehensive and accurate brain information, revealing correlations between different modes of BN” what does mode mean here?

Reply: The mode here refers to the different modes of brain networks constructed, including functional networks, white matter structural networks and gray matter structural networks.

3)Add a citation for Montreal Cognitive Assessment (MoCA)..Also typo in reference 27.

Reply: Reference 27 label has been moved to Montreal Cognitive Assessment (MoCA).

4)What is b0 image? what is FN? What is HMR?

Reply: b0 image: A ‘b0 image’ is an image taken when no diffusion-weighted gradient is applied, also known as a baseline image or an image without diffusion-weighted.

FN: The number of fiber bundles.

HMR: The hypergraph manifold regularization term.

5)Reference for “A deterministic fiber bundle tracking algorithm was employed, with tracking parameters set as anisotropy index < 0.15 and tracking angle < 45° to calculate indicators such as FN between brain regions”?

Reply: Reference has been added.

6)Section 2.3.3, references missing for first 6 lines.

Reply: A reference has been added.

7)C is correlation matrix and also point edge association matrix?

Reply: Yes, it's been changed to remove ambiguity.

8)Figures are of poor quality...so please include better quality ones

Reply: Figures that have been processed with PACE have been uploaded.

**Minor comments**

1)Check English in below sentences.

a) It offers a powerful representation of the patterns of interaction between different regions of brain.

Reply: "It" has been changed to "BN".

b) We are able to obtain information that can identify individual participants.

Reply: This sentence has been deleted.

c)Numerous other typos and grammatical errors also exists. please check the entire paper.

Reply: The entire paper has been checked and amended appropriately.

**Review Comments to the Author**

Reviewer #1:

1. Compared with the existing state-of-the-art methods, what are the core innovations in your work?

Reply: a) For the first time, an individual-based gray matter network specific to ESRDaMCI was constructed using KV extracted from DKI; b) The application of a multi-head self-attention mechanism is a novel approach to model ESRDaMCI's brain function network, as well as the fusion network of white matter structure and gray matter structure; c) By utilizing the aforementioned fusion network, this study successfully identifies discriminative brain regions associated with the pathogenesis of ESRDaMCI. Furthermore, it analyzes the changes in topological attributes of these discriminative brain regions and identifies brain regions that significantly contribute to cognitive dysfunction.

2. The Abstract should be more concise.

Reply: The Abstract has been simplified appropriately.

3. There are too many processes and methods introduced in section 2.1 and 2.2, and these are not your main work.

Reply: Parts have been deleted.

4. The attached figures look a little blurry and needs to be replaced with a clearer version.

Reply: Figures that have been processed with PACE have been uploaded.

Reviewer #2:

1. Reference for sentence “Epidemiological surveys indicate that cognitive dysfunction is highly prevalent among ESRD patients, with conservative estimates suggesting that 16% to 38% of them experience cognitive impairment.”

Reply: It has been modified.

2. In my opinion the sentence “Mild cognitive impairment (MCI) serves as an intermediate stage between normal cognitive function and cognitive dysfunction.” could be improved to “Mild cognitive impairment (MCI) represents the intermediate stage between the normal decline in memory and thinking associated with aging and the more pronounced cognitive deterioration observed in dementia.”

Reply: It has been modified to "Mild cognitive impairment (MCI) represents the intermediate stage between the normal decline in memory and thinking associated with aging and the more pronounced cognitive deterioration observed in dementia".

3. Add references in the sentence “These techniques include functional magnetic resonance imaging (fMRI) [6], structural magnetic resonance imaging [7], magnetic resonance spectroscopy [8], fluorodeoxyglucose positron emission tomography [9], single photon emission tomography [10], arterial spin labeling [11], diffusion tensor imaging [12], and diffusion kurtosis imaging (DKI) [13]”

Reply: We already have one reference for each technique, so we shouldn't need to add any more.

4. The sample sizes of the two groups are not well balanced. There are much fewer controls than MCI subjects.

Reply: Patients with mild cognitive impairment were easier to identify and recruit into the study, while the control group was relatively difficult to recruit because they typically did not actively seek medical help or participate in relevant studies. In studies involving medical or health information, the control group had higher expectations and demands for privacy protection, which may have influenced their willingness to participate. The study focused on patients with mild cognitive impairment, so the sample size of the patient group may be relatively larger to better understand and analyze this specific group.

5.In the discussion the authors should add the strengths.

Reply: There are advantages to writing in the first paragraph of the discussion: “Compared to conventional fusion networks, our proposed model's self-attention mechanism can…thereby allowing for further exploration of the associations between multiple modalities.”

Reviewer #3:

1.The title of the figures should not appeared separately in the manuscript.

Reply: It has been deleted.

2.Figure legends should be added and listed at the end of the References.

Reply: It has been added.
